# IL-33/ST2 Axis in Organ Fibrosis

**DOI:** 10.3389/fimmu.2018.02432

**Published:** 2018-10-24

**Authors:** Ourania S. Kotsiou, Konstantinos I. Gourgoulianis, Sotirios G. Zarogiannis

**Affiliations:** ^1^Department of Respiratory Medicine, Faculty of Medicine, School of Health Sciences, University of Thessaly, BIOPOLIS, Larissa, Greece; ^2^Department of Physiology, Faculty of Medicine, School of Health Sciences, University of Thessaly, BIOPOLIS, Larissa, Greece

**Keywords:** epithelial cells, inflammation, Interleukin-33, myofibroblasts, organ fibrosis, ST2, tissue remodeling

## Abstract

Interleukin 33 (IL-33) is highly expressed in barrier sites, acting via the suppression of tumorigenicity 2 receptor (ST2). IL-33/ST2 axis has long been known to play a pivotal role in immunity and cell homeostasis by promoting wound healing and tissue repair. However, it is also involved in the loss of balance between extensive inflammation and tissue regeneration lead to remodeling, the hallmark of fibrosis. The aim of the current review is to critically evaluate the available evidence regarding the role of the IL-33/ST2 axis in organ fibrosis. The role of the axis in tissue remodeling is better understood considering its crucial role reported in organ development and regeneration. Generally, the IL-33/ST2 signaling pathway has mainly anti-inflammatory/anti-proliferative effects; however, chronic tissue injury is responsible for pro-fibrogenetic responses. Regarding pulmonary fibrosis mature IL-33 enhances pro-fibrogenic type 2 cytokine production in an ST2- and macrophage-dependent manner, while full-length IL-33 is also implicated in the pulmonary fibrotic process in an ST2-independent, Th2-independent fashion. In liver fibrosis, evidence indicate that when acute and massive liver damage occurs, the release of IL-33 might act as an activator of tissue-protective mechanisms, while in cases of chronic injury IL-33 plays the role of a hepatic fibrotic factor. IL-33 signaling has also been involved in the pathogenesis of acute and chronic pancreatitis. Moreover, IL-33 could be used as an early marker for ulcer-associated activated fibroblasts and myofibroblast trans-differentiation; thus one cannot rule out its potential role in inflammatory bowel disease-associated fibrosis. Similarly, the upregulation of the IL-33/ST2 axismay contribute to tubular cell injury and fibrosis via epithelial to mesenchymal transition (EMT) of various cell types in the kidneys. Of note, IL-33 exerts a cardioprotective role via ST2 signaling, while soluble ST2 has been demonstrated as a marker of myocardial fibrosis. Finally, IL-33 is a crucial cytokine in skin pathology responsible for abnormal fibroblast proliferation, leukocyte infiltration and morphologic differentiation of human endothelial cells. Overall, emerging data support a novel contribution of the IL-33/ST2 pathway in tissue fibrosis and highlight the significant role of the Th2 pattern of immune response in the pathophysiology of organ fibrosis.

## Introduction

Interleukin (IL) 33 was first described in 1999 as a protein (DV27) overexpressed in vasospastic cerebral arteries in a canine subarachnoid hemorrhage model (1). Later, in 2003, it was characterized at the molecular level as a nuclear factor abundantly expressed in human high endothelial venule cells (2). The mechanisms mediating its effects were elucidated by Schmitz et al., as well as Dinarello, that identified IL-33 as a cytokine of the IL-1 superfamily (3, 4). Its name was given due to its adherence to the style of the IL-1 superfamily nomenclature, indicating that IL-33 was not merely a functional copy of IL-1α and IL-1β proteins (3, 4). The role of IL-33 as an alarmin was established participating in tissue homeostasis, signaling via the IL-1 receptor-related suppression of tumorigenicity 2 receptor (ST2), and inducing T helper type 2 (Th2) immune responses (3, 4).

Normally human IL-33 is mainly expressed and stored in the nucleus of endothelial and epithelial cells (5). IL-33 is a dual-function cytokine: the full-length IL-33 protein (flIL-33) serves as an intranuclear gene regulator, and the mature IL-33 (mIL-33) serves as an extracellular cytokine upon release from damaged or necrotic cells (6). IL-33 is passively and rapidly released from damaged cells, as a tissue-barrier component in response to stimuli or cell injury. However, it can also be actively secreted by immune cells. The abundant basal IL-33 expression in tissues can be further increased during inflammation (7). Notably, it has been documented that the fraction of IL-33 produced by tissues rather than that provided by immune cells, is necessary for Th2-induced airway inflammation (8). An inflammatory microenvironment may exacerbate disease-associated functions of IL-33 through the generation of highly active mature forms (9). Neutrophil serine proteases such as cathepsin G and elastase which secreted during inflammation have been shown to regulate IL-33 activity, by processing flIL-33 and generate biologically highly active mature forms of IL-33, *in vivo* (10). Furthermore, serine proteases secreted by activated mast cells (chymase and tryptase) generate mIL-33 with potent activity on lymphoid cell type 2 ILC2s (11). On the contrary, it is still unknown whether and which endogenous proteases have a similar capacity (7). It has only been reported an endogenous calcium-dependent caspase which is called calpain that mediates pro-IL-33 cleavage and mIL-33 production. Calpain is secreted when the cells are severely damaged by external stimulation such as inflammatory stimuli; and subsequently, the level of intracellular calcium ion is raised by an influx of extracellular ion or a release from an intracellular store (7, 12). Although both flIL-33 and mIL-33 can bind to and signal through ST2, mIL-33 exhibit 10-fold higher affinity and bioactivity than flIL-33 (6, 10).

Conversely, the ST2 receptor is predominantly expressed by immune cells involved in innate immunity, including mast cells, ILC2s, macrophages, dendritic cells (DCs), eosinophils, basophils, natural killer cells (NK cells). Furthermore, ST2 is expressed by cells participating in adaptive immunity such as CD4 +, CD8 + T cells, and T-regulatory cells (Tregs) (13, 14).

In humans, there are three ST2 isoforms. IL-33 signals via the ST2L receptor which has a membrane-bound domain, an extracellular segment composed of three linked immunoglobulin-like motifs, and a cytosolic Toll/interleukin-1 receptor domain. The soluble ST2 (sST2) isoform lacks the transmembrane and cytoplasmic domains and includes a unique nine amino-acid C-terminal sequence, constitutes a decoy receptor that does not signal. The ST2V isoform which is characterized by the absence of an immunoglobulin-like motif and alternative splicing of the C-terminal portion of ST2 is thought to be a form which is primarily found in gastrointestinal tissues (15, 16).

The IL-33/ST2 axis has been widely studied in respiratory, digestive, urogenital, heart and liver pathologies and the abundance of literature suggests a pivotal role of this pathway in the pathogenesis of an increasing number of diseases (Table 1). Emerging data have shown that IL-33/ST2 axis is involved in a variety of biological processes such as the development and regulation of immune responses, restoration of normal tissue homeostasis by promoting wound healing and repair. However, the IL-33/ST2 signaling pathway is involved in the loss of balance between extensive inflammation and tissue regeneration lead to remodeling that constitutes the hallmark of fibrosis (14, 72).

**Table 1:** The main roles of IL-33 in organ fibrosis.

**Fibrotic disease**	**IL-33 sources**	**Role of IL-33**	**IL-33/ST2 Axis**	**References**
**PULMONARY FIBROSIS**				
IPF	Airway epithelial cells Endothelial cells Fibroblasts Myofibroblasts Innate immune cells	Pro-fibrogenetic role	*mIL-33:* ST2-, Th2-, and macrophage-dependent fashion	(17, 18)
SSc-related fibrosis				(19)
BLM-induced fibrosis				(6, 20–23)
			*flIL-33:* ST2-independent, Th2-independent fashion	
**LIVER FIBROSIS**				
Cirrhosis	Hepatocytes Hepatic stellate cells	Pro-fibrogenetic role	ST2-, Th2-, ILC2s-dependent fashion	(20, 24–28)
Viral hepatitis				(29)
Primary biliary cirrhosis				(30, 31)
NASH				(32, 33)
**PANCREATIC FIBROSIS**				
Acute and chronic pancreatitis Pancreatic cancer	Pancreatic stellate cells Pancreatic myofibroblasts Pancreatic acinar cells	Pro-inflammatory role Pro-fibrogenetic role	ST2- dependent fashion Proliferation and migration of pancreatic myofibroblasts	(18, 34–37)
Murine autoimmune pancreatitis Human IgG4-related AIP	Plasmacytoid dendritic cells	Pro-inflammatory role Pro-fibrogenetic role	ST2- dependent fashion	(38)
**INTESTINE FIBROSIS**				
IBD (UC Pediatric Crohn's ileitis)	Colonic subepithelial myofibroblasts Stromal cells Epithelial cells Pericryptal fibroblasts	Pro-inflammatory role Pro-fibrogenetic role	ST2-, Th2- dependent fashion	(39–47)
**RENAL FIBROSIS**				
Transplanted kidney interstitial fibrosis	Peritubular capillary endothelial cells Human kidney cells	Pro-inflammatory role Pro-fibrogenetic role Renal tubular Epithelial to Mesenchymal Transition	ST2-, Th2-, ILC2s- dependent fashion	(48–51)
IRI-induced renal fibrosis				(52–54)
Diabetic kidney disease SLE, SSc associated Kidney fibrosis				(19, 55, 56)
**HEART FIBROSIS**				
Heart failure Myocardial infarction Post-implantation	Cardiac fibroblasts Cardiomyocytes	Cardioprotective effects Anti-hypertrophic effects Anti-fibrotic effects	ST2-, Th2- dependent fashion	(57–64)
**SKIN FIBROSIS**				
Cutaneous fibrosis Irradiation-induced fibrosis	Dermal fibroblasts	Pro-fibrogenetic role Pro-inflammatory role Morphologic differentiation of human endothelial cells	Eosinophil-mediated Th2 immune responses ST2-, Th2- Tregs-dependent fashion	(3, 65–67)
SSc-associated fibrosis				(19, 68–70)
BLM-induced skin fibrosis				(71)

*BLM, Bleomycin; EMT, Epithelial to Mesenchymal Transition; flIL-33, full-length Interleukin 33; IBD, Inflammatory Bowel Disease; IL-33, Interleukin 33; ILC2s, Innate Lymphoid Cell type 2; IPF, Idiopathic Pulmonary Fibrosis; IRI, Ischemia-Reperfusion Injury; mIL-33, mature Interleukin 33; NASH, Non-alcoholic Steatohepatitis; SLE, Systemic Lupus Erythematosus; SSc, Systemic Sclerosis; ST2, Suppression of Tumorigenicity 2; Th2, T helper type 2; UC, Ulcerative Colitis*.

Despite the burden of human organ fibrosis, there are still many things unknown regarding the underlying mechanisms. On this note, it has been supported that the IL-33/ST2 axis exerts anti-inflammatory and anti-proliferative effects in many diseases; however, it has also been shown that results in fibrotic effects in others. Although several lines of evidence demonstrate that there is a potential role of IL-33/ST2 in remodeling and differentiation processes in, there is still room for better understanding. The aim of this review is to critically evaluate the available evidence regarding the role of the IL-33/ST2 axis in organ fibrosis.

## IL33/ST2 axis participates in the TH2-mediated inflammatory response and exacerbates tissue remodeling

Acute wounds initiate an early inflammatory response, directing the subsequent phases of healing (73). However, the triggers of inflammation and wound repair upon injury are still unclear (73). IL-33 has been found to participate in the early inflammatory process as other members of the IL-1 family do (73). More specifically, increased mRNA and protein expression after scratching *in vivo* have been demonstrated; suggesting a role for IL-33 in wound healing (74). Furthermore, ST2 receptor signaling improves wound closure by promoting the transition of macrophages from an inflammatory to a non-inflammatory state during healing, supporting epidermal closure, angiogenesis, and reduced scarring (73). During wound healing extracellular mIL-33 interacts with the ST2L receptor and the complex of IL-33/ST2L activates myeloid differentiation primary response 88 (MyD88) intracellular cascades that drive production of type 2 cytokines (such as IL-13) from polarized Th2 cells (73). Besides, it has been suggested that proteolytically uncleaved, flIL-33, which remains predominantly intracellular and intranuclear, promotes inflammation in an ST2-independent fashion through regulation of gene expression (75).

The key role of the IL-33/ST2 axis in tissue remodeling is better understood considering the crucial role of immune responses during tissue regeneration. Of note, increased production of epithelial IL-33 could lead to accumulation of innate type 2 cells during the alveolar period of lung development; that is when the lung is maximally remodeled (76). IL-33 promotes ILC2s function that enhance tissue healing, remodeling, and homeostasis in the post-partum period. More convincingly, studies in adult murine models showed that IL-33 activates lung ILC2s and renders them resistant to interferon-γ (IFN-γ)-mediated suppression of IL-5 and IL-13 production (22). Th2 cytokines such as IL-13 are crucial mediators of inflammation and remodeling (22). In addition, IL-33 activates alternatively activated M2 macrophages that control tissue remodeling during lung postnatal branching morphogenesis (77).

In other words, type 2 immunity influences lung development and/or remodeling while the spontaneous activation of type 2 cells in the embryonic period have been found to require extracellular IL-33 and ST2 signaling (76, 78). Meanwhile, IL-33 contributes to the development of type 2 immune environment in lungs at a young age as it lowers the threshold for innate immune responses to allergens (76).

## IL-33/ST2 axis in pulmonary fibrosis

Pulmonary fibrosis is a non-neoplastic pulmonary disease that is primarily caused by an uncontrolled wound-healing response. Idiopathic pulmonary fibrosis (IPF) is a highly lethal pathological entity of unknown etiology that is characterized by inflammation, fibroblast accumulation, and excessive collagen deposition. Importantly, IL-33 mRNA and protein levels have been found significantly increased in the bronchoalveolar lavage (BAL) fluids of patients with IPF (17) and systemic sclerosis (SSc)-related fibrosis as compared to healthy controls (19). Furthermore, the expression of IL-33 mRNA was also enhanced in IPF lung tissue (23).

IL-33 is also elevated in the bleomycin (BLM)-induced murine model of lung injury and fibrosis (20, 21, 23). The lungs of BLM-treated mice showed a substantial accumulation of IL-33-positive cells (20, 21, 23). Specifically, it has been documented that IL-33 and BLM result in synergistic effects on pulmonary fibrosis *in vivo* (6). In detail, mIL-33 production is induced in macrophages by BLM (6, 20, 21, 23). Subsequently, IL-33 enhances the polarization of macrophages toward an M2 phenotype (6, 79, 80). It is well established that the pro-fibrogenic activity of IL-33 is mainly attributed to its involvement in M2 macrophage polarization, as macrophages with alternative activation, rather than classical activation, serve to accelerate pulmonary fibrosis (6, 79, 80). A clear link between biologic splice variants of IL-33 (mIL-33) and a Th2 innate immune response has been demonstrated (80). Mature forms of IL-33 have been reported to drive production of extremely high levels of Th2 cytokines such as IL-13 (80). Furthermore, M2 macrophages are polarized by IL-13, and they further promote a Th2 reaction through the IL-13 and the transforming growth factor β (TGF-β) production, vice versa (20–23).

Several studies have demonstrated the importance of Th2 cells in fibrosis since IL-4, IL-5, and IL-13 have been causally linked to fibrosis (80). In detail, IPF fibroblasts are hyper-responsive to cytokines such as IL-13, at the same time, fibroblasts and innate immune cells are important sources of IL-33 (80). Enhanced production of TGF-β and IL-13 are essential for the development of pulmonary fibrosis by inducing myofibroblast differentiation and stimulating the production of extracellular matrix components, such as collagen (79, 80). Therefore, these data support that precise control of alveolar TGF-β activation and IL-13 are essential for alveolar homeostasis (79).

Previous work has shown that deficiency of the Akt2 isoform resulted in M2 macrophages polarization producing IL-13 and TGF-β and in the expansion of IL-13 recruiting ILC2s (6). Thus, Akt2 regulates pulmonary fibrosis by up-regulating the pro-fibrotic TGF-β and IL-13 production by macrophages (81). It is likely that the induction of IL-13 precedes and is essential for the subsequent enhanced M2 macrophage polarization by IL-33 (6). Moreover, it has been shown that in response to IL-33 treatment, Akt2^−/−^macrophages displayed decreased production of IL-13 and TGF-β1 and attenuated phosphorylation of transcription factor Forkhead box O3a (FoxO3a) to stop acting as a trigger for apoptosis (81). Inhibition of Akt2 marked as a potential strategy for treating IPF (81).

Interestingly, BLM can also induce fIL-33 secretion from airway epithelial cells and alveolar macrophages in an ST2-independent, Th2-independent fashion, likely through cytokine regulation of several non-Th2 cytokines, such as TGF-β, IL-6, and monocyte chemoattractant protein-1 (MCP-1) and possibly by engaging heat shock protein (HSP) 70 (6). flIL-33 can then be processed into various mature forms of IL-33 by neutrophil proteases. mIL-33 subsequently stimulate macrophages and ILC2s to produce IL-13 (75). On the other hand, this response may be mediated only by nuclear-located fIIL-33 affecting gene expression. Collectively, these data suggest that flIL-33 is also potentially implicated in the pulmonary fibrotic process (6, 75).

ST2 is mainly expressed in endothelial and type II alveolar epithelial cells as well as innate immune cells such as macrophages and ILC2s in the lungs. sST2 levels were also found increased in serum and BAL of acute exacerbation of IPF and BLM-induced lung fibrosis, respectively (82). ST2 mRNA expression has been reported to be increased in the BLM-induced lung fibrosis model *in vivo*, as well as in a human lung fibroblast and a human type II alveolar epithelial cell line, possibly reflecting the development of a type 2 pattern of inflammatory process in the fibrotic lung tissue (82, 83).

Li et al. reported that ST2 deficiency or administration of an anti-IL-33 antibody were able to attenuate bone marrow (BM)-induced pulmonary fibrosis (6). Similarly, intranasal administration of a lentivirus for epithelial over-expression of sST2 has been reported to attenuate pulmonary fibrotic change by inhibiting the expression of pro-inflammatory and pro-fibrotic mediators, such as IL-13, IL-33, and TGF-β1 along with improved survival rates in BLM-treated mice (20).

Increased numbers of lung ILC2s have also been implicated in BLM-induced fibrosis in the mouse (6). Especially, BM-derived ST2-expressive ILC2s have been recently reported to be recruited to the fibrotic lung through the IL-33/ST2 pathway and contributed to fibroblast activation perhaps via transforming TGF-β (84). Furthermore, adoptive ILC2 transfer into recipient mice enhances lung fibrosis, whereas blocking IL-33 or ST2 deficiency diminish fibrosis. The increase in IL-33 responsive ILC2s is not unique to animal models of pulmonary fibrosis since they are also increased in SSc, correlating with the extent of fibrosis. ILC2s are also present in the IPF lung tissue and BAL, wherein they are associated with upregulated expression of lung IL-33.

Even in cases of asthma, experimental results showed that direct murine airway exposure to IL-33 could induce local fibrotic changes. IL-33/ST2 axis is thought to at least partially mediate the fibroblast function and local expression of matrix metalloproteinases (MMPs) and their inhibitors as well as other fibrosis-related proteins (85). Additionally, IL-33 is probably related to prostaglandin E2 (PGE2) production, stimulating mast cells to produce a large quantity of PGE2 demonstrating potent anti-fibrotic activity in the IPF lung (17, 86). IL-33 can regulate deposition of extracellular matrix (ECM) and promote the process of pulmonary fibrosis by inducing the imbalance between MMP9 and tissue inhibitor of MMP9 (TIMP-1) (17, 86).

Finally, fibulin-1 (Fbln1), an important ECM component involved in a matrix organization and wound repair, has been found to predict disease progression in IPF patients (87). Genetically inhibited Fbln1 has been associated with reduced levels of pro-inflammatory cytokines such as IL-33 and pulmonary inflammatory cells in a murine COPD model (87).

Hence, IL-33 is thought to be a novel cytokine that promotes the initiation and progression of pulmonary fibrosis by recruiting and directing inflammatory cell function and enhancing pro-fibrogenic cytokine production in an ST2- and macrophage-dependent manner (6, 79, 80). However, fIL-33 secretion from airway epithelial cells and alveolar macrophages acts in an ST2-independent, Th2-independent fashion in the fibrotic process. The over-expression of sST2 decoy receptor or any other exogenous inhibition of IL-33/ST2L signaling result in markedly lower levels of IL-33 and other pro-inflammatory and pro-fibrotic mediators thus attenuate the fibrotic process.

## IL-33/ST2 axis in liver fibrosis

Liver fibrosis is a reversible wound healing response to acute or chronic hepatocellular injury from various etiologies, including viral infection, cholestasis, metabolic diseases, and alcohol abuse. It has been suggested that damage associated molecular patterns (DAMPs) act as molecular links between hepatocyte death and liver fibrogenesis (27). Evidence indicates that when acute and massive liver damage occurs, the release of IL-33 by injured hepatocytes might act as an activator of tissue-protective mechanisms, while in cases of chronic injury IL-33 plays the role of a hepatic fibrotic factor (27). For instance, melatonin which exerts cytoprotective effects via inhibition of oxidative stress and apoptosis in liver ischemia-reperfusion injury (IRI) has been proposed to inhibit liver fibrosis through suppressing necroptotic DAMPs signaling cascades, such as the IL-33 signaling pathway (88).

IL-33/ST2 axis has been implicated in several hepatic diseases, such as cirrhosis, virus infection, fatty liver disease and toxic liver damage leading further to liver fibrosis (24, 27–29). Indeed, IL-33 has shown potential liver fibrosis promoting effect (24, 32). It has been found that in murine and human fibrotic livers, IL-33 levels as well as the mRNA expression of both IL-33 and ST2, are higher as compared to healthy liver (24, 28). Their expression is significantly increased along with the severity of fibrosis, especially in cirrhotic livers (33). Likewise in patients with primary biliary cirrhosis, an autoimmune liver disease that could result in liver failure, and hepatoma carcinoma, the serum IL-33 levels were positively correlated with disease severity (30, 31).

The role of ST2 has also been highlighted in liver fibrosis. It has been documented that liver injury, inflammatory cell infiltration, and fibrosis are reduced in the absence of the receptor ST2L (24). Furthermore, it has been found that ST2L deficient mice did not increase collagen production when challenged with carbon tetrachloride, an organic compound with pro-fibrotic effects (24, 28). Similarly, the absence of ST2L prevented liver inflammation both in the acute and chronic phases, with attenuated activation of mitogen-activated protein kinase (MEK)\extracellular signal-regulated kinase(ERK)/p38- mitogen-activated protein kinase (MAPK) signaling cascade(25, 232).

Besides, sST2 has been regarded as a circulating biomarker to reflect IL-33 activation and fibrosis in patients with liver diseases. In fact, sST2 serum levels differ between hepatitis B virus-infected patients were dependent on the severity of hepatic fibrosis (89). Especially, the plasma levels of sST2 were found to be associated with mortality in patients with HBV-related acute-on-chronic liver failure (89). Notably, in a murine liver fibrosis model, the antibody blockade of sST2 enhances the severity of fibrosis (89).

It has been reported that the major source of IL-33 in fibrotic livers is the hepatic stellate cells (HSCs) that have also been suggested to be the leading producers of ECM proteins (25, 27, 32). Injury-associated immunological processes supporting trans-differentiation of quiescent HSC to fibrogenic myofibroblasts in the course of liver injury are particularly important in fibrosis (90). Moreover, an ST2 expression has been observed on the membrane of HSCs (32). However, other data derived from murine and human studies demonstrated that hepatocytes are the primary sources of IL-33 both in the fibrotic liver and in healthy liver (27, 32). More specifically, IL-33 release as a DAMP upon hepatocyte damage may have a direct effect on HSCs that increases secretion of cytokines and production of collagen (27, 32). Besides, another study showed that activation of HSCs was decreased in ST2-deficient liver fibrosis mice (24). IL-33-mediated Th2 immune response promotes HSCs proliferation, TGF-β synthesis, and fibrogenesis (27). As in lung tissue, Th2 pro-fibrotic cytokines production such as IL-4, IL-5, and IL-13 are known to play a critical role in liver fibrosis (27). On the other hand, Th1 cytokines lead to a rapid and intense inflammatory response while causing little fibrosis.

Interestingly, vector-encoded overexpression of IL-33 was sufficient to induce fibrosis in the liver without administration of chemicals, demonstrating the pro-fibrotic role of IL-33, predominantly exerted through the IL-13 induction (27). In particular, IL-13 could initiate activation and differentiation of HSCs by enhancing TGF-β signaling through IL-4Rα and signal transducer and activator of transcription 6 (STAT6) in HSCs, promoting liver fibrosis (28). Some other data suggest that IL-13, rather than TGF-β, primarily activates HSCs in liver fibrosis (32). Furthermore, the IL-33/ST2/IL-13 pathway is thought to be Galectin-3 (Gal-3) dependent (91–93). Gal-3 has been found to attenuate steatosis while promoting liver injury, inflammation and fibrosis in an obesogenic mouse model of non-alcoholic steatohepatitis (NASH) (91–93). Therefore, Gal-3 inhibitors have been suggested to protect against fibrotic disorders (91, 92).

More convincingly, the stimulation of *in vitro* activated HSCs with recombinant IL-33 (rIL-33) induced the MAPK pathways that were found to be mediated by ERK, Jun N-terminal kinase (JNK) and p38 protein kinases (32). Moreover, HSCs activated by rIL-33 *in vitro*, released IL-6, TGF-β, and resulted in the stimulation of α-smooth muscle actin (a-SMA) and collagen expression (32). These data suggest a direct fibrogenic role of IL-33 in HSCs, which is potentially synergistic with its effects on ILC2s (26, 28). Hence, another mechanism proposed to be involved in liver fibrosis is through the activation of ILC2s via the ST2 signaling pathway, resulting again in a release of several Th2 cytokines (30, 33, 94). IL-33 induces the activation and expansion of ILC2s to express IL-13 and IL-5, which subsequently causes M2 macrophage and eosinophil accumulation and regulates ST2^+^ Tregs homeostasis in liver adipose tissue through attenuating adipose tissue inflammation (95–97). In fact, the upregulation of IL-33 was positively correlated with an increase of ILC2s (32, 98). Activated ILC2-derived IL-13 initiated activation and differentiation of HSCs via the IL-4Rα-STAT6 transcription factor-dependent pathway, as previously described (28).

Non-alcoholic Fatty Liver Disease (NAFLD) which comprises simple steatosis, NASH, cirrhosis and possibly liver carcinoma, is potentially related to a severe form of the fibrotic liver disease; however, how fat deposition renders hepatocytes susceptibility to inflammatory, lipid and oxidative stress mediators is still unidentified. In obesity, immune cells infiltrating the visceral adipose tissue mediate chronic low-grade inflammation that plays a critical role in the pathogenesis of NAFLD (99). It has been demonstrated that administration of rIL-33 aggravates liver fibrosis in an ST2-dependent manner during experimental NAFLD, which is further shown by a substantial reduction of experimentally-induced liver fibrosis in mice lacking IL-33 (28, 33).

In NASH, through secreted cytokines, intrahepatic innate and adaptive immune cells sustain chronic inflammation and induce trans-differentiation of HSCs into myofibroblasts, which are critical cells for liver fibrosis (90). Remarkably, IL-33 treatment has been proposed to attenuate diet-induced hepatic steatosis on the one hand, but aggravate hepatic fibrosis in an ST2-dependent manner on the other hand (33). These findings provide evidence for a dual role of the IL-33/ST2 axis in diet-induced NASH in mice. Similarly, additional results were recently obtained showing that injury-induced endogenous IL-33 release is sufficient to cause inflammation and fibrosis in the bile duct ligated mouse model, which is not further enhanced by rIL-33 (32). More contradictory data reported that IL-33 deficiency in mice does not lessen liver fibrosis during diet-induced steatohepatitis (100).

Hence, in liver fibrosis, evidence indicate that when acute and massive liver damage occurs, the release of IL-33 might act as an activator of tissue-protective mechanisms, while in cases of chronic injury IL-33 shows a significant liver fibrosis promoting effect in an ST2-, Th2- dependent fashion across the entire spectrum of liver pathology.

## IL-33/ST2 axis in ancreatic fibrosis

Pancreatic fibrosis is one of the characteristic histopathological findings in cases of chronic pancreatitis. The fibrosis develops as a result of abnormal activation of stromal cells and deposition of ECM proteins. Identification of essential regulators of pancreatic fibrosis, mainly the pancreatic stellate cells (PSCs), has contributed significantly to the understanding of the cellular and molecular basis of these pathogenic processes (101). There is accumulating evidence that PSCs play a key role in the development of pancreatic fibrosis in chronic pancreatitis and pancreatic cancer (34, 102). Additionally, IL-33 is a novel factor involved in the pathogenesis of chronic pancreatitis and possibly pancreatic cancer. In addition, IL-33 has been found to exacerbate acute pancreatic (AP) inflammation in mice (35).

IL-33 is expressed in the nucleus of activated PSCs. Baseline IL-33 expression was reported to be low in quiescent rat PSCs but increased upon cellular activation with mediators such as IL-1b, tumor necrosis factor a (TNF-a), lipopolysaccharide (LPS), platelet-derived growth factor (PDGF)-BB (36). In detail, IL-1b induces IL-33 expression via activation of the nuclear factor (NF)-kβ and ERK pathways and partially through p38 MAPK, whereas PDGF-BB induces IL-33 expression primarily via activating the ERK signaling pathway (18).

It has been recently proposed that IL-33 induction is associated with the transformation to an α-SMA positive PSCs myofibroblastic phenotype. However, treatment of PSCs with rIL-33 did not stimulate any specific phenotype, while a reduction of IL-33 expression resulted in decreased proliferation of PSCs in response to PDGF-BB. Pancreatic myofibroblasts responded to IL-33 by the expression of pro-inflammatory mediators, and increased proliferation and migration, thus playing a crucial role in the progression of pancreatic fibrosis (103). Vice versa, pancreatic myofibroblasts express and secrete modest levels of IL-33 mRNA and protein, respectively. Expression of the ST2 was detected in PSCs and pancreatic myofibroblasts (36).

Moreover, Watanabe et al. found that IL-33 secretion by pancreatic acinar cells under the influence of type I IFN plays a significant role in the development of pancreatic fibrosis occurring in a model of conventional pancreatitis (38). Furthermore, substance P released by pancreatic acinar cells was shown to synergize IL-33 and augment mast cell activation that subsequently regulates the release of several inflammatory mediators in the initiation and progression of AP (35). Remarkably, a triangular link between the cytokine IL-33, pancreatic acinar cells, and mast cells in the development and progression of AP exists (35, 37). Recently Leema G et al. investigated the protective effects of scopoletin, a coumarin compound with anti-inflammatory activities on AP and associated lung injury in mice and found an anti-inflammatory effect by down-regulating substance P signaling via Nf-κB pathway (104).

Besides, activation of plasmacytoid dendritic cells (pDCs) producing IFN-α and IL-33 plays a pivotal role in the chronic fibro-inflammatory responses underlying murine autoimmune pancreatitis (AIP) and human IgG4-related AIP (38).

Watanabe et al. also suggested the possibility that microbe-associated molecular patterns act as pDC activators in AIP, indicating that this form of pancreatic inflammation is initiated and/or driven by gut bacterial components (38). However, further studies defining the gut microbiome in AIP, as well as the demonstration that gut bacteria are translocated into the circulation and can thus contact pancreatic cells, will be required to fully establish this concept (38).

Therefore, IL-33 is considered to be a novel factor implicated in the pathogenesis of acute and chronic pancreatitis and potentially in tissue fibrosis. Specifically, the expression of the ST2 in PSCs, pancreatic myofibroblasts, and pDCs implying a role for IL-33 signaling within the pancreas in an autocrine, and/or paracrine fashion.

## IL-33/ST2 axis in intestinal fibrosis

IL-33/ST2 axis seems to represent an important mediator in intestinal fibrosis. Normal epithelium and stroma of the intestine express large amounts of IL-33 and ST2 during the homeostatic turnover of the intestinal mucosa (39). It has been demonstrated that intestinal baseline IL-33 expression was present in pericryptal fibroblasts and was increased during infection (105). A role for IL-33/ST2 signaling in the differentiation of stem cells in organoid culture was also elucidated (105).

IL-33 has been associated with areas of compromised barrier function and plays a critical role in maintaining normal gut homeostasis (44). Uncontrolled IL-33 expansion potentially leads to barrier dysfunction of epithelium, chronic relapsing inflammation, and fibrotic lesions (41–43). Additionally, IL-33 induces enteric glia to secrete glial cell-line derived neurotrophic factor family ligands (GFLs) that play an essential role in intestinal epithelial barrier homeostasis by maintaining tight junctions and negatively regulating local inflammatory response (106, 107). Moreover, IL-33 influences the enteric nervous system to induce intestine hypermotility t to expel invading parasites from the intestine, while is an important regulator of the gut microbiome (108, 109).

Within the gut mucosa, colonic subepithelial myofibroblasts SEMFs are primary sources of IL33, particularly in ulcerated lesions from patients with ulcerative colitis (UC) and induce potent Th2 immune responses (45, 46). The localization of IL-33-producing SEMFs in mucosal ulcerations suggests a significant role of the cytokine in wound healing. Actually, it could be used as an early marker for ulcer-associated activated fibroblasts and myofibroblasts trans-differentiation. Overall, one cannot rule out the potential role of IL-33 in gut-associated fibrosis, particularly in the setting of turnover of chronic tissue damage and repair, characteristics of inflammatory bowel disease (IBD) (45, 46).

The IL-33 expression is enhanced specifically in inflamed mucosa in UC, while exogenous IL-33 treatment in mice, modulated higher colonic mucin release (45, 46). Moreover, IL-33 mRNA levels have been associated with UC disease activity (18, 44, 47). Sponheim et al. found that a feature of IBD-associated mRNA IL-33 expression is the accumulation of both fibroblasts and myofibroblasts in UC lesions. In ulcerations, the fibroblast marker HSP47, platelet-derived growth factor receptor (PDGFR) β, and in part the SEMFs marker α-SMA were expressed (46). Epidermal growth factor has also been demonstrated to contribute to increased IL-33 production and ST2 expression (110).

Epithelial-IL-33 was also increased in pediatric Crohn's ileitis and strongly associated with clinical and histopathological findings, ileal eosinophilia, and complicated fibrostenotic disease (40). Furthermore, neutralization of IL-33 interferes with the massive influx of eosinophils into the gut mucosa and potently decreases fibrogenic gene expression and fibrosis (111).

sST2 constitutes a marker of IBD severity, found to be significantly increased in both the gut mucosa and the serum in both patients and experimental models of IBD. However, in IBD patients, ST2L mRNA expression remained similar to that of healthy controls (14, 44).

IL-33 promotes ILC2s in the gut to produce the growth factor amphiregulin (AREG) that binds to the epidermal growth factor receptor (EGFR) which are responsible for tissue repair during inflammation and restoration of mucosal integrity (42, 112). Disruption of the AREG/EGFR signaling pathway is involved in human patients and murine models of IBD (113, 114). On the other hand, IL-33 stimulation of ILC2s could be beneficial in intestinal inflammation outside the setting of infection for instance in cases of helminth intestinal infection (115). ATP released by parasite-infected cells stimulates local mast cells to produce IL-33, which then activates IL-13-producing ILC2s necessary for helminth expulsion (115).

The role of the IL-33 is not limited to Th2 responses but could also amplify Th1-mediated inflammation (41). IL-33 is an activator of Tregs, activation of which appears to be a compensatory mechanism for intestinal inflammation (112, 116). Colonic Tregs preferentially express ST2. Signaling through ST2/IL-33 promotes both Treg accumulation and maintenance in the gut, enhancing their protective function (117).

The role of IL-33 in colitis as well as in colon cancer is controversial, being very dependent on the timing of alarmin activation or expression relative to the damaging insult (41, 118). Regarding the colon cancer, on the one hand, it has been supported that IL-33 promotes an IFN-γ-mediated immune protective mechanism that helps guard against the development of sporadic colon cancer that links to inflammation (39). IL-33 inhibits colon cancer growth by suppressing cellular proliferation and promoting apoptosis (119). Interestingly, IL-33 has been found to potently induce a neutralizing ant-IL-13 receptor that plays an important protective role in cases of mucosal damage (120–122). Conversely, Zhang et al., found that tumor-derived IL-33 following the activation of tumor cells by pro-inflammatory cytokines such as IL-13, modulates the tumor microenvironment to potently promote colon carcinogenesis and liver metastasis in murine models (123). Furthermore, IL-33 stimulation of human colonic SEMFs has been found to induce the expression of growth factors associated with intestinal tumor progression and extracellular matrix components (124). Similarly, experimental studies in a murine model showed that overexpression of IL-33 promoted the expansion of ST2+Tregs, increased Th2 cytokine milieu, and induced M2 macrophages in the gut, thereby increasing tumor development (125). Furthermore, lower expression of ST2L has been reported in human colon tumors. ST2L expression was negatively associated with the higher tumor grade (126). Inhibition of the IL-33/ST2 pathway may limit mucositis and thus improve the effectiveness of chemotherapy (127).

It is ambiguous if IL-33 is a consequence of intestinal inflammation or if IL-33 is a critical instigator in promoting an inflammatory response. Taken together, there is clear evidence that IL-33/ST2 axis participates in maintaining normal gut homeostasis, particularly in promoting mucosal wound healing and repair. When deregulated, this important ligand-binding pair can also play a critical role in the progression of chronic inflammation and fibrosis, leading to gastrointestinal-related disorders such as IBD as well as colon cancer.

## IL-33/ST2 axis in renal fibrosis

Renal fibrosis is characterized by progressive connective tissue expansion through kidney parenchyma, leading to detrimental renal function deterioration (128, 129). Almost all the cell types in the kidneys participate in the pathogenesis of renal fibrosis, illustrating the complexity of this process (48, 51, 52). Specifically, a study by Manetti et al. found that nuclear IL-33 expression in the fibrotic kidneys of patients with SSc was absent in the endothelial cells of peritubular capillaries while ST2 has been expressed abundantly in kidney glomeruli, tubules, and peritubular capillaries (19). Conversely, in control human kidney (HK) specimens, IL-33 was found to be constitutively expressed in peritubular capillary endothelial cells (49). The levels of IL-33 and sST2 were relevant to the progressive deterioration of kidney function while a significant correlation between the serum level of sST2 and disease severity has been shown (50).

Epithelial to Mesenchymal Transition (EMT) of podocytes, tubular epithelial cells, and circulating fibrocytes which are transformed to mesenchymal fibroblasts migrating to adjacent interstitial parenchyma constitutes the principal mechanism of renal fibrosis (129, 130). Other key events in tubulointerstitial fibrosis that also take place in the glomeruli after injury are glomerular infiltration of inflammatory cells and myofibroblastic activation of the mesangial cells (129, 131–133).

Emerging evidence supported that the renal tubular EMT is a remarkable process in the pathogenesis of renal interstitial fibrosis, mediated by IL-33 (50). IL-33 has been found to participate in transplanted kidney interstitial fibrosis promoting EMT of HK-2 cells in a dose- and time-dependent manner, via the activation of the p38 MAPK signaling pathway (129). These data suggest that therapies are targeting a reduction in IL-33 levels or on the downregulation of the p38 MAPK signaling axis may be an effective strategy in the prevention of kidney interstitial fibrosis (129).

IL-33 is also a marker of IRI that contributes to innate immune cell recruitment and development of renal graft damage associated with renal transplantation in humans (53). Furthermore, it plays a significant role in the pathogenesis of IRI-induced renal fibrosis through regulating myeloid fibroblast accumulation, inflammatory cell infiltration, and cytokine and chemokine expression (52, 53). Th2- cytokines, including IL-4, IL-5, and IL-13, all induced by IL-33, play an essential role in the renal fibrotic disease (52, 53).

Experimental data revealed that IL-33-treated IRI mice had increased levels of IL-4 and IL-13 in serum and renal tissue as well as more ILC2s, Tregs, and anti-inflammatory M2 macrophages, as compared to control-treated IRI mice (54). Furthermore, it has been reported that depletion of ILC2s substantially abolished the protective effect of IL-33 on renal IRI (54). Conversely, adoptive transfer of *ex vivo*-expanded ILC2 prevented renal injury in mice subjected to IRI. Besides, treatment of mice with IL-33 or ILC2 after IRI had a protective effect associated with induction of M2 macrophages in kidney and required the ILC2 production of amphiregulin (54). Interestingly, mice treated with sST2 exhibited less severe renal dysfunction and fibrosis in response to IRI compared with vehicle-treated mice (54). Furthermore, inhibition of IL-33 suppressed BM-derived fibroblast accumulation and myofibroblast formation in the kidneys after IRI stress, which was associated with less expression of ECM proteins (54). Hence, IL-33 signaling in ILC2s plays a critical role in the pathogenesis of IRI–induced renal fibrosis and treatment with IL-33 inhibitor reduced pro-inflammatory cytokine and chemokine levels in the kidneys of mice following IRI insult (54).

Furthermore, it is increasingly recognized that episodes of acute kidney injury (AKI) increase the susceptibility of chronic kidney disease (CKD) and end-stage renal disease (ESRD) that are characterized by organ fibrogenesis (134, 135). It has been found that the administration of rIL-33 exacerbated cisplatin-induced AKI by acting as a pro-inflammatory cytokine (49). In a cisplatin-induced mouse AKI model, IL-33 was reported to promote AKI through CD4+ T cell-mediated production of chemokine (C-X-C motif) ligand (CXCL) 1, which could exacerbate the renal damage. In addition, high expression levels of IL-33 have been observed in LPS-induced acute glomerular injury (49, 136).

Additionally, IL-33 released from necrotic cells has been implicated in autophagy, which can balance increased apoptosis secondary to contrast-induced nephropathy in diabetic kidney disease (55). Another study also reported that IL-33 contributes to kidney fibrosis associated with systemic lupus erythematosus (SLE).

Thus, emerging data indicate that the upregulation of the IL-33/ST2 signaling pathway may promote tubular cell injury and fibrosis predominantly via EMT in the kidneys (56).

## IL-33/ST2 axis in heart fibrosis

Heart failure (HF) and cardiac fibrosis are associated with IL-33 mainly in cases of a mechanical strain of cardiac fibroblasts (57, 64). IL-33 demonstrates anti-hypertrophic and anti-fibrotic effects on cardiomyocytes, transduced by ST2L. In 2007, Sanada et al. first documented that IL-33 prevents cardiomyocyte apoptosis, reduces infarct size, fibrosis, and apoptosis through induction of anti-apoptotic proteins after ischemia-reperfusion in rats and improves cardiac function and survival after myocardial infarction (57). IL-33 correlated with the expression kinetics of the anti-apoptotic gene B-cell lymphoma 2 (Bcl-2), which is in agreement with its anti-apoptotic role (58). Thereafter, multiple experimental studies have also illustrated that IL-33 attenuates cardiac fibrosis induced by the increased cardiovascular load, showing that IL-33 directly inhibits pro-fibrotic activities of cardiac fibroblasts (58, 59, 137). Treatment of rat cardiac fibroblasts with IL-33 was also found to impair the migratory activity of fibroblasts or their precursors into the stressed myocardium (57, 138). IL-33 levels were found to be significantly elevated upon a cyclic stretch of cardiac fibroblasts *in vitro*, and the administration of IL-33 was shown to inhibit myocyte amino acid incorporation and growth thus protecting against cardiac hypertrophy (57). IL-33 protected cardiomyocytes from hypoxia-induced apoptosis *in vitro*, and this effect was partially inhibited by sST2, highlighting the critical role of IL-33 in regulating cardiac myocyte function and its protective role in cardiac fibrotic diseases (58).

Others have reported that ablation of IL-33 gene caused exaggerated cardiac remodeling in both ischemic and non-ischemic HF, It leads to cardiomyocyte hypertrophy and cardiac fibrosis upon mechanical stress, impaired cardiac function, and survival (60). Furthermore, it has been recently shown that IL-33 acts by reducing a form of erythrocyte superoxide dismutase (eSOD) production, thus eSOD is found decreased in chronic HF (61). eSOD is a protective enzyme against oxidative stress in chronic HF (61). Alternatively, the *in vitro* administration of IL-33 significantly decreased cardiac interstitial fibrosis in wild-type mice underwent transaortic constriction surgery to increase cardiovascular load (57).

Of note, the aforementioned IL-33 benefits were absent in mice with deletion of the ST2 gene, so these data indicate that IL-33 exerts its cardioprotective role only through the ST2 receptor signaling. Moreover, the microRNA-587b has been proposed to ameliorate cell apoptosis, inflammatory reaction of myocarditis, and fibrosis through inhibition of the IL-33/ST2 pathway by suppressing IL-33 (139).

In contrast, sST2 disrupts the cardioprotective effects of IL-33 by sequestering its availability for binding with the transmembrane receptor ST2L. sST2 has been demonstrated as a marker of myocardial fibrosis and HF progression. Both cardiac fibroblasts and cardiomyocytes express IL-33 and sST2, and expression levels are increased as a response to myocardial stress (15). This issue is supported from a clinical perspective, given that sST2 concentrations have repeatedly been found high in patients with acute myocardial infarction and acute HF and correlate with parameters of infarct magnitude, cardiac dysfunction, hemodynamic impairment, and neurohormonal derangement (15). Based on the above sST2 is thought to be a biomarker for poor outcome in patients with cardiovascular disease (140–142). Moreover, cardiogenic shock and increased C-reactive protein levels are associated with higher sST2 levels. The PRIDE (Pro-Brain Natriuretic Peptide Investigation of Dyspnea in the Emergency Department) study highlighted the potential applications of sST2 in acute HF (143). Thereafter, several studies that followed emphasized on its diagnostic and prognostic utility (144–146). sST2 may also identify patients who benefit most from cardiac resynchronization therapy defibrillators (147), titration of beta blockers (62) and angiotensin-converting enzyme inhibitors (148). Weir et al. showed that sST2 could predict functional recovery and left ventricle remodeling during the post-infarction period (149). The sST2 levels were positively correlated with the degree of cardiac fibrosis (150). Along these lines, it has been recently observed that left ventricular assist device (LVAD) resulted in a significant drop in sST2 levels with normalization within 3 months post-implantation, thus lessened heart fibrosis and inflammation (151).

According to the above-mentioned studies, recently, a highly sensitive ELISA for sST2 (Presage ST2) as well as a rapid quantitative lateral flow immunoassay for measurement of sST2 in human plasma has been developed, allowing for point-of-care testing (142). The first one was approved by regulatory agencies both in Europe and the United States for prognostication in HF. The second one (Aspect-PLUS ST2 test, Critical Diagnostics, San Diego, CA, USA) has received regulatory approval in Europe, but it has yet to be approved by the Food and Drug Administration in the United States (142). The American College of Cardiology Foundation/American Heart Association (ACCF/AHA) guidelines of 2013 have incorporated sST2 as a relevant marker of fibrosis. They recommend it for additive risk stratification in patients with acutely decompensated HF (level of evidence A) or chronic HF (level of evidence B) (63).

Additionally, the metabolic activity of epicardial adipose tissue has been recently associated with a decrease in the IL-33 levels, thus was closely related to the development of cardiac fibrosis at 1-year post-myocardial infraction (150).

Moreover, Gal-3 has been associated with left ventricular remodeling along with an increased risk of incident HF and mortality (152). It has been reported that Gal-3 promotes myocardial fibrosis, whereas myocardial fibrosis and hypertrophy are prevented through interaction between IL-33 and sST2 (57).

Hence, IL-33 demonstrates cardioprotective, anti-hypertrophic and anti-fibrotic effects on cardiomyocytes, transduced by ST2L, and disturbing by sST2.

## IL-33/ST2 axis in skin fibrosis

IL-33 is released by dermal fibroblasts (3). It has been documented that the IL-33/ST2 signaling is associated with abnormal fibroblast proliferation, leukocyte infiltration and morphologic differentiation of human endothelial cells, resulting in increased endothelial permeability, consistently with increased angiogenesis and ECM deposition *in vivo* (65).

IL-33 has been reported to be a crucial signaling cytokine in skin pathology by inducing IL-13-dependent cutaneous fibrosis mechanism, required both eosinophils and recombination-activating gene (RAG)-dependent lymphocytes. Eosinophils contribute to tissue remodeling and fibrosis (65). It is known that in skin diseases, eosinophil expresses a broad spectrum of Th2 cytokines such as IL-4, IL-5, IL-13, and C-C motif chemokine-11 (CCL11/eotaxin) (66). The different cytokine expression patterns suggest distinct functional roles of eosinophils in various diseases that might be related to host defense, immunomodulation, fibrosis, and/or tumor development (67).

More convincingly, in cutaneous fibrosis, the injection of rIL-33 induces collagen production via ST2-dependent recruitment of BM-derived eosinophils that further secrete IL-13 in response to IL-33 stimulation (22, 65). IL-33 has also been proposed as a critical molecule operating in eosinophil-mediated fibrosis in the high-dose-per fraction irradiated skin (66). In detail, vascular endothelial cells damaged by high-dose radiation secrete IL-33, which may stimulate fibrotic responses via eosinophil recruitment and eosinophil-mediated Th2 immune responses.

Rankin et al. demonstrated that IL-33 induces cutaneous fibrosis and intense inflammation that are associated with large numbers of CD3+ cells and F4/80+ myeloid cells, except infiltrating eosinophils (22). Additionally, IL-33 was also shown to induce several others cytokines such as IL-4, IL-5, tissue inhibitor of metalloproteinases 1 (TIMP1), MMP12, and MMP13 gene expression; however, it did not induce the expression of TGF-β1 which participated with varying degrees in fibrogenesis (22).

As far as SSc is concerned the most severe variant is characterized by aggressive skin fibrosis (68). These patients experience a low health-related quality of life that is directly related to the extent of the dermal fibrosis (68). Serum levels of IL-33 are elevated in SSc, even more pronounced in diffuse cutaneous SSc than in limited cutaneous SSc; thus these levels are positively correlated with the total fibrotic skin score. In other words, serum IL-33 levels are likely to reflect the degree of endothelial damage in patients with SSc (68).

IL-33 might mediate very early pathogenic events of SSc through recruitment and stimulation of ST2-expressing cells (immune cells and fibroblast/myofibroblast) (68). Other studies reported that increased circulating levels of IL-33 in SSc correlate with early disease stage and microvascular involvement being a serum marker for vascular abnormalities in SSc (69). IL-33 induces migration of Th2 lymphocytes and enhances Th2 cytokine production. Remarkably, owing to the Th2-type signature, there are elevated levels of both IL-4 and IL-13 in SSc patient's sera (67), while SSc patients exhibit substantial Th2 cytokine production in cultures of CD4+ T lymphocytes isolated from their affected skin (153). It is likely that the signaling mechanism in the dermal fibroblasts mediated by IL-13 is STAT6 (67, 154, 155).

A recent study demonstrated that the rs7044343 polymorphism of the IL-33 gene was associated with susceptibility to SSc in a Turkish population. No similar studies were found in the literature (156).

Conversely, sST2 constitutes a potential marker for disease progression in limited cutaneous SSc with disease duration over 9 years. On the contrary, sST2 was not elevated in healthy controls or SSc patients with early skin involvement or disease duration shorter than 9 years (157). Furthermore, sST2 serum levels were lowered by iloprost (prostacyclin) treatment (157). The question remains why sST2 is elevated in limited cutaneous SSc and not in diffuse cutaneous SSc patients. Diffuse cutaneous SSc and limited cutaneous SSc may be two pathophysiologically different diseases rather than two subtypes of one disease showing significant differences of organ involvement, disease progression and significantly different chemokine levels between two entities (158, 159). Accordingly, sST2 could be a marker for pathological alterations and higher sST2 in limited cutaneous SSc could be partially beneficial by blocking the high inflammatory capacity of IL-33 by neutralizing its bioactivity.

In addition, evidence is presented to support the high tissue-localized expression of IL-33 in patients with SSc, as well as IL-33-dependant skin-localized Tregs trans-differentiation into Th2-like cells, combined with expression of the ST2 receptor on Tregs. In other words, IL-33 might be an important stimulator of tissue-localized loss of normal Tregs function (70). Moreover, friend leukemia virus integration 1 (Fli1) -a predisposing factor of SSc- haploinsufficiency increases Th2- and Th17-like Tregs proportions in BLM-induced pro-fibrotic skin condition, in which IL-33-producing dermal fibroblasts contribute to Th2-like Tregs trans-differentiation (71).

Consequently, IL-33 is a crucial cytokine in skin pathology responsible for abnormal fibroblast proliferation, leukocyte infiltration and morphologic differentiation of human endothelial cells, leading to fibrotic skin conditions.

## Conclusion

In this review, we spotlight the distinctive contribution of IL-33/ST2 signaling in organ fibrosis as well as the significant role of the Th2 pattern of immune response in the pathophysiology of organ fibrosis. The IL-33/ST2 axis widely participates in the fibrotic process of many vital organs, demonstrating clear direct effects on wound healing and remodeling. Generally, the IL-33/ST2 signaling pathway has mainly anti-inflammatory/anti-proliferative effects. However, chronic tissue injury is responsible for pro-inflammatory/pro-fibrogenetic responses. At the basal level, both flIL-33 and mIL-33 forms have been reported to contribute to fibrogenesis. The axis influences the capacity of various cells to trans-differentiate into extracellular matrix-secreting activated myofibroblasts which constitute the main cell population of fibrosis, in an organ-specific underlying mechanism. Furthermore, the IL-33/ST2 axis is involved in angiogenesis, production of matrix components, ECM deposition. Importantly, elevated levels of IL-33 and/or sST2 constitute markers of dysfunction and severity in many fibrotic diseases. IL-33/ST2 axis seems to be a promising therapeutic target in fibrosis constitutes, therefore, a critical area for further investigation.

## Author contributions

OK took part in decision on structure and content of the review, performing literature, search, and writing the review. KG revised the draft critically; gave final approval of the submitted version. SZ took part in decision on structure and content of the review, contributed to writing the review and gave thorough feedback throughout the process, and accepting the final version.

### Conflict of interest statement

The authors declare that the research was conducted in the absence of any commercial or financial relationships that could be construed as a potential conflict of interest.

## Abbreviations

AP: acute pancreatitis
ASCF: American College of Cardiology Foundation
AHA: American Heart Association
Acute Kidney Injury
ALI: Acute Lung Injury
AP-1: Activator Protein 1
a-SMA: α-Smooth Muscle Actin
BAL: Bronchoalveolar Lavage
BLM: Bleomycin
BM: Bone Marrow
CKD: Chronic Kidney Disease
CXCL: Chemokine (C-X-C motif) Ligand
DAMPs: Damage Associated Molecular Patterns
ECM: Extracellular Matrix
EGFR: Epidermal Growth Factor Receptor
EMT: Epithelial to Mesenchymal Transition
ERK, Extracellular Signal-Regulated Kinase, eSOD: Erythrocyte Superoxide Dismutase
ESRD: End-Stage Renal Disease
Fbln1: fibulin-1
Fli1: Friend Leukemia Virus Integration
flIL-33: full-length Interleukin 33
FoxO3a: Forkhead box O3a
Gal-3: Galectin-3
GFLs: glial cell-line derived neurotrophic factor family ligands
HF: Heart Failure
HK: Human Kidney
HSCs: Hepatic Stellate Cells
HSP: Heat Shock Protein
IBD: Inflammatory Bowel Disease
IL: Interleukin
IL-13Ra1: IL-13 Receptor subunit alpha 1
ILC2s: Innate Lymphoid Cell type 2
IFN-γ: Interferon-γ
IPF: Idiopathic Pulmonary Fibrosis
IRI: Ischemia-Reperfusion Injury
JNK: Jun N-terminal kinase
LPS: Lipopolysaccharide
LVAD: Left Ventricular Assist Device
MAPK: Mitogen-Activated Protein Kinase
MCP-1: Monocyte Chemoattractant Protein-1
MEK: Mitogen-Activated Protein Kinase\ERK kinase
mIL-33: mature Interleukin 33
MMP: Matrix Metalloproteinase
MyD88: Myeloid Differentiation primary response 88
NAFLD: Non-alcoholic Fatty Liver Disease
NK cells: Natural Killers cells
NASH: Non-alcoholic Steatohepatitis
NF: Nuclear Factor
PDGF: Platelet-Derived Growth Factor
PDGFR: Platelet-Derived Growth Factor Receptor
pDCs: plasmacytoid Dendritic Cells
PGE2: Prostaglandin E2
PRIDE: Pro-Brain Natriuretic Peptide Investigation of Dyspnea in the Emergency Department
PSCs: Pancreatic Stellate Cells
RAG: Recombination-Activating Gene
SEMFs: Subepithelial Myofibroblasts
SLE: Systemic Lupus Erythematosus
SSc: Systemic Sclerosis
STAT6: Signal Transducer and Activator of Transcription 6
ST2: Suppression of Tumorigenicity 2
sST2: soluble ST2
ST2L: transmembrane ST2
TGF-β: Transforming Growth Factor β
Th1: T helper type 1
Th2: T helper type 2
TIMP1: Tissue Inhibitor of Metalloproteinases 1
TNF-a: Tumor Necrosis Factor a
Tregs: T-regulatory cells
rIL-33: recombinant Interlrukin-33
UC: Ulcerative Colitis.
